# Macrophage-specific deletion of Notch-1 induced M2 anti-inflammatory effect in atherosclerosis via activation of the PI3K-oxidative stress axis

**DOI:** 10.18632/aging.205342

**Published:** 2023-12-26

**Authors:** Mingming Zhang, Xiangyong Yue, Xueping Zhao, Yonggang Lu, Hongtao Liu, Zhe Zhang, Huan Ma, Xing Wang, Hanying Xing

**Affiliations:** 1 Clinical Medicine Research Center, Hebei General Hospital, Shijiazhuang, Hebei 050051, China; 2 Department of Oncology, Hebei General Hospital, Shijiazhuang, Hebei 050051, China; 3 Department of Nursing, Hebei General Hospital, Shijiazhuang, Hebei 050051, China; 4 Clinical Laboratory, Hebei General Hospital, Shijiazhuang, Hebei 050051, China

**Keywords:** Notch-1, atherosclerosis, PI3K/AKT, exosome, Notch-1^Mac-KO^

## Abstract

Objective: Notch-1 signaling is significantly associated with the occurrence and development of atherosclerosis (AS). However, the molecular mechanisms underlying the specific deletion of Notch-1 in AS-associated macrophages are not fully understood. This study aimed to investigate the effects of Notch-1 in AS.

Methods and Results: Tissue samples were obtained from atherosclerotic segments of human carotid arteries. Immunofluorescence staining showed that Notch-1 was significantly colocalized with macrophages (CD68+), and Notch-1 staining was increased in human vulnerable plaques. Notch-1^MAC-KO^/ApoE^−/−^ mice were generated in which Notch-1 was selectively inactivated in macrophages, and WT for littermate control mice (ApoE^−/−^/Notch-1^WT^). A control group was then established. All mice fed with a high-fat and Oil Red O, Movat, a-SMA, CD68, and Sirius red staining were used to evaluate the morphology. Specific deletion of Notch-1 in macrophages repressed the pathophysiology of AS. Immunofluorescent staining and Western blotting revealed that Notch-1^MAC-KO^ repressed M1 and M2 responses in AS. Here, GSEA revealed that Notch-1 activation and PI3K signaling were statistically significantly correlated with each other, and Notch-1 was involved in the regulation of the PI3K signaling pathway. In the *in vitro* experiments, the secretion of Arg-1 and exosomes was classified by peritoneal macrophages of Notch-1^MAC-KO^/ApoE^−/−^ and Notch-1^WT^/ApoE^−/−^ mice. Immunohistochemistry staining and Western blotting were used to measure the expression levels of Notch1, PI3K, p-PI3K, AKT, p-AKT, Arg-1, IL-6, CD36, SREBP-1, CD206, iNOS, cleaved-caspase-3/-9, Bax, CD9, Alix and TSG101 in the peritoneal macrophages and exosomes, respectively.

Conclusions: The specific deletion of Notch-1 in macrophage represses the formation and development of AS via the PI3K/AKT signaling pathway.

## INTRODUCTION

Atherosclerosis (AS) is a chronic inflammatory disease of the vascular wall, which contributes to high morbidity and mortality of cardiovascular diseases [1, 2]. It is an arterial disease associated with multiple risk factors such as obesity, high-saturated fat diets, hypercholesterolemia, hypertension and aging [3–5]. During AS formation, inflammation-derived endothelial dysfunction is the first step, followed by the recruitment of inflammatory cells (macrophage and T cells), the degeneration of extracellular matrix, and the disruption of elastic lamina and collagen networks, which weakens the arterial wall and ultimately results in the formation of atherosclerotic plaques [6–8].

Although many types of cells are involved in the development and progression of AS, macrophages play a fundamental role. They mainly exist in two functionally distinct phenotypes, classically activated or M1 macrophages (pro-inflammatory) and alternatively activated or M2 macrophages (anti-inflammatory) [9, 10]. Accumulation of macrophages in the artery wall is a prominent feature of atherosclerotic plaques. M1 macrophages predominate in the unstable plaques, whereas the fibrous cap encompassing the necrotic lipid core contains both M1 and M2 macrophages [11]. Macrophage phenotype polarization has been well recognized in AS, but the underlying mechanism remains elusive.

Notch is a family of single-pass transmembrane receptors. The Notch signaling pathway is highly conserved in mammals and is involved in a wide variety of life activities such as development and tissue homeostasis [12]. As a member of the Notch receptor family, Notch homolog 1 (*Notch-1*) is involved in inflammation-mediated AS as a key regulator of chronic inflammation [13]. Previous studies have shown that inhibiting the Notch-1 signaling pathway with Notch-1 inhibitor DAPT or siRNA in cultured human monocytes increased M1 macrophage population [14]. However, the effects of macrophage Notch-1 signaling *in vivo* remain undetermined. The present study examined Notch-1 expression in human atherosclerotic lesions and investigated the effects of macrophage-specific deletion of Notch-1 on macrophage phenotype polarization and AS in mice.

To investigate the downstream signaling molecules of Notch-1, bioinformatics analysis was performed using AS datasets downloaded from the GEO database to determine the association between Notch-1 and PI3K/AKT pathway, a pathway crucial for macrophage polarization [15–17]. Furthermore, *in vivo* experiments were performed to verify the impact of macrophage-specific deletion of Notch-1 on vascular PI3K/AKT activity, and an anti-PI3K neutralizing antibody LY-294002 was employed to confirm the mediation role of PI3K in Notch-1-derived M2 polarization.

## MATERIALS AND METHODS

### Collection of human carotid segments from AS patients

Human samples were obtained from Hebei General Hospital. The samples and data of patients are used under the full protection of the privacy of patients (Table 1). The samples used in this study are discarded after routine clinical treatment, which has no impact on the diagnosis and treatment of patients. No additional examinations or tests will be performed for the patients due to their participation in the study, which will not cause harm to the patients. Therefore, an exemption from informed consent was applied for and approved by the Hospital Ethics Committee before the study. After successful anesthesia, EEG, somatosensory evoked potential and cerebral blood flow TCD were monitored in a supine position. The head rest was used to fix the head and fully expose the affected side of the neck. The incision about 6 cm long was made along the front of the affected side of the neck. The surgical area was routinely disinfected with iodine and sterile surgical drapes were laid. The skin, subcutaneous tissue and platysma muscle were cut along the marked line, and the carotid triangle was bluntly separated along the medial side of sternocleidomastoid muscle. The tissue continued to be peeled deep along the inner edge of sternocleidomastoid muscle to the carotid sheath, the tissue was cut around the carotid sheath, the common carotid artery was carefully separated, exposed, and dissociated upward to the bifurcation of the internal carotid artery, so as to fully expose the common carotid artery, internal carotid artery, external carotid artery and superior thyroid artery. Lidocaine was injected into the arterial wall at the bifurcation of the common carotid artery. After the common carotid artery, external carotid artery and internal carotid artery were clamped in turn, the cerebral blood flow monitoring showed that the ipsilateral MCA blood flow decreased significantly, close to the baseline, with bypass indication. The common carotid artery and internal carotid artery were cut off, and the intraoperative bypass system was inserted into the common carotid artery and internal carotid artery. The temporary clamp was removed, normal saline was injected into the balloon, and the bypass tube was fixed. The shunt was successful (temporary occlusion for about 3 minutes and transient recovery of cerebral blood flow). The hyperplastic intima and plaque were removed (the surgical area was repeatedly washed with heparin saline during dissection and resection), the common carotid artery stump was cleaned, the internal carotid artery stump was treated, and the plaque was removed and sent for pathological examination. The arterial wall was tightly sutured with 7-0 gel vascular suture. The bypass tube was removed and the internal carotid artery was opened. After the blood vessel was opened, cerebral blood flow monitoring showed that the blood flow of ipsilateral MCA increased (less than 100%) as compared to that before. The common carotid artery was temporarily blocked again, followed by moderate reduction of blood pressure, and it was opened after the blood flow was stable. The wound was covered with instant gauze to stop bleeding. After no active bleeding was detected, the carotid sheath was sutured, a subcutaneous drainage tube was placed outside the sheath, and finally the muscles, subcutaneous tissues and skin were sutured layer by layer.

**Table 1:** Clinicopathological characteristics of AS patients.

**Characteristics**	**Stable group**	**Vulnerable group**
Age	65.84 ± 4.125 *N* = 5	68.41 ± 5.43 *N* = 5
Gender	Male: 4/Female: 1	Male: 4/Female: 1
Hyperlipidemia	*N* = 5	*N* = 5
Diabetes	*N* = 2	*N* = 3
Hypertension	*N* = 1	*N* = 2
Maximum diameter under ultrasound (mm)	1.121 ± 0.125 *N* = 5	3.692 ± 0.1543 *N* = 5^**^

^**^P < 0.01, Stable group vs. Vulnerable group.

### Construction of Notch-1^MAC-KO^ and ApoE^−/−^/Notch-1^MAC-KO^ mice

Floxed Notch-1 mice (Notch-1 flox/flox, LoxP sites were inserted on both sides of the exon 3–4 of Notch-1 gene to construct floxed Notch-1 mice) and mice expressing the Cre recombinase under the control of the lysozyme 2 (Lyz2) promoter (Lyz2-Cre; both purchased from the JAX Laboratory) were used to construct myeloid-specific Notch-1 knockout (Notch-1^MAC-KO^) mice (knockout of the Notch-1 gene exon 3–4) [18]. Mouse genotyping was carried out using a standard protocol described in the JAX Genotyping Protocol Database. To generate mice with a double knockout of ApoE and Notch-1 (ApoE^−/−^/Notch-1^MAC-KO^), and littermate control (ApoE^−/−^/Notch-1^WT^), Notch-1^MAC-KO^ and Notch-1^WT^ mice were crossed with ApoE^−/−^ mice, respectively. A control group was then established. Male mice at 6–8 weeks of age were used in the present study.

### Mouse model of AS

All mice were housed at room temperature of (22 ± 2°C), relative humidity of 40–60% and 12 h light/dark cycle. ApoE^−/−^/Notch-1^WT^ mice were defined as W.T. control group; ApoE^−/−^/Notch-1^MAC-KO^ mice as K.O. group, 20 mice per group. A high-fat diet was used to induce AS. On day 28, the mice were anesthetized with sodium pentobarbital (40 mg/kg) and sacrificed through cervical dislocation. All animal procedures were approved by the Animal Experiment Committee of Hebei General Hospital in accordance with the Guide for the Care and Use of Laboratory Animals published by the US National Institutes of Health (8th Edition, 2011).

### Acquisition and culture of mouse peritoneal macrophages

Peritoneal macrophages were prepared and cultured as described previously [19]. Briefly, the mice were fixed in a supine instantly after euthanasia, the abdominal skin was disinfected with 70% ethanol, a small incision was made to peel the skin off and expose the peritoneal wall, 7 mL of cold phosphate-buffered saline (PBS) was injected into the peritoneal cavity, the abdomen was gently massaged, and the fluid was withdrawn, followed by centrifugation at 400 g for 5 minutes. The cell pellet was collected. Then the cells were resuspended and plated in 24-well culture plates at a cell density of 1 × 10^6^ cells/mL. After 24 h, the cells were rinsed with PBS, counted, and processed for further experiments.

### Macrophage cell line culture

The mouse macrophage cell line RAW264.7 was cultured in Dulbecco’s modified Eagle’s medium (DMEM) supplemented with 10% FBS, 100 U/ml pen/strep, 1 × nonessential amino acids (NEAA) and streptomycin 100 U/mL, and cultured at 37°C in a 5% CO_2_ humidified atmosphere. The cells were incubated with LPS (1 μg/mL) and ox-LDL (10 μg/mL) overnight at 37°C respectively to induce an inflammatory response. At the time points indicated, the supernatants and cell lysates were collected and stored in 1 mL aliquots at −70°C for further analyses.

### Western blotting

The protein levels of p-PI3K (Abcam, ab182651, 1:1000), PI3K (Abcam, ab191606, 1:1000), p-Akt (Abcam, ab81283, 1:10000), Akt (Abcam, ab179463, 1:10000), Arginase-1 (Abcam, ab124917, 1:1000), CD206 (Abcam, ab64693, 1:1000), iNOS (Abcam, ab178945, 1:1000), IL-6 (Abcam, ab259341, 1:1000), cleaved-caspase-3 (Abcam, ab214430, 1:5000), caspase-9 (Abcam, ab184786, 1:1000), Bax (Abcam, ab263897, 1:1000), CD36 (Abcam, ab252922, 1:1000), SREBP-1 (Abcam, ab313881, 1:1000), Alix (Abcam, ab186429, 1:1000), TSG101 (Abcam, ab133586, 1:1000) and CD9 (Abcam, ab307085, 1:1000) were examined by Western blotting. First, descending aortic tissue and peritoneal macrophages were lysed in RIPA buffer containing protease and phosphatase inhibitors on ice. Second, the protein concentration was measured using a BCA protein assay kit. Third, the total proteins were separated on 5% SDS-PAGE and transferred onto the nitrocellulose membrane. The membrane was then blocked with 15% non-fat milk at room temperature for 1.5 h, and then incubated with the primary antibodies (Abcam) at 4°C for 24 h. Finally, the bands were visualized by an enhanced chemiluminescence kit (ECL; Amersham Pharmacia Biotech, Piscataway, NJ, USA). The assay was repeated three times.

### Immunohistochemistry and double immunofluorescence staining assay

Histological and morphological examinations were performed as previously described [7]. Immunohistochemical staining was performed to examine Notch-1, CD68 (a marker for macrophages) and α-SMA (a marker for activated fibrotic cells) in AS tissues. The aorta segments (carotid artery and thoracic aorta) from AS patients or mice were dissected, fixed in 10% formalin, embedded in Optimum Cutting Temperature Medium (OCT) and sectioned into 5 μm-thick slices with a cryostat, followed by staining with primary antibodies against Notch-1 (Abcam, USA), CD68 (Abcam) or α-SMA (Abcam), and horseradish peroxidase (HRP)-labeled secondary antibodies. Then they were visualized by 3,3′-diaminobenzidine (DAB) chromogenic reaction and counterstained with hematoxylin. In double immunofluorescence staining, the sections were incubated with mouse anti-CD68 (ab955), Arginase-1 (D4E3M™) XP^®^ Rabbit mAb #93668 and Rabbit monoclonal (E6) to Notch1 (ab245686) primary antibodies at 4d incubated co-incubated 3 times and then incubated with Goat Anti-Mouse IgG H&L (Alexa Fluor^®^ 647) (ab150115), Donkey Anti-Rabbit IgG H&L (Alexa Fluor^®^ 488) (ab150073) and Goat Anti-Rabbit IgG H&L (Alexa Fluor^®^ 488) (ab150077) at room temperature away from light for 45 minutes. Immunofluorescent monostain was performed using Mouse monoclonal (NOS-IN) to iNOS (ab49999) as the primary antibody and Goat Anti-Mouse IgG H&L (Alexa Fluor^®^ 647) (ab150115) as the secondary antibody. Then the image was acquired under a fluorescence microscope. All data were analyzed by Image-Pro Plus software version 5.1 (Media Cybernetics, Rockville, MD, USA).

### Movat staining and Sirius red staining for aortic roots and oil red O staining for descending aorta

After fixation with paraffin, heart sections were exposed to alcoholic dehydration, incubated with 0.1% Sirius red solution for 50 min, and sliced into 5 μm-thick sections. The sections were randomly assigned to conduct Movat pentachrome staining and oil red O staining. Movat pentachrome staining was conducted using the above method [20, 21]. Computer aided morphometry was used to determine the cross-sectional area of the five-color stained sections of Movat. Sirius red staining was performed for 1 h. Finally, the nuclei were stained with Mayer hematoxylin solution for 10 min, and the sections were sealed with neutral gum and analyzed with a polarization microscope. Sirius red staining was used to assess the collagen levels in atherosclerotic lesions. After longitudinal dissection of descending aorta, oil red O working solution was added to the arteries for 10 minutes. Then, 6% isopropyl alcohol was used to wash away the working fluid and the sections were sealed with glycerin gelatin. The total surface area and ORO-positive lesion area were measured using NIS-element software. The total plaque size was measured on Movat pentachrome-stained specimens, and the lipid deposition area was measured on oil red O-stained specimens. Representative images were evaluated by Image-Pro Plus software (open the image in the software, click Measure-count/size, select colors, select the red area in the image in the automatic area, click close after selection, and finally click count) [19, 22].

### Serum cytokine ELISA assay

The levels of mouse serum IL-6, TNFα, IL-10 and Arg-1 were measured using commercially available ELISA kits (Abcam, USA). Standard and serum samples were prepared and assayed according to the manufacturer’s instructions for the corresponding ELISA kit. The concentrations of IL-6, TNFα, IL-10 and Arg-1 were expressed as pg/mL and ng/L. The assay sensitivity was less than 2 pg/mL for both IL-6, TNFα, IL-10 and Arg-1. There was no cross-reactivity with any other cytokines present in the serum sample.

### Statistical analysis

Statistical significance between two groups was assessed with unpaired Student’s *t*-test. One-way ANOVA was performed for comparisons among groups, and SAS 9.0 software (SAS Institute Inc., Cary, NC, USA) was used. All values were represented as mean ± SEM. *P* < 0.05 was considered statistically significant.

### Data availability

The data that support the findings of this study are available from the corresponding author upon reasonable request.

## RESULTS

### Increased Notch-1 and iNOS expressions in human vulnerable atherosclerotic lesions

**Figure 1:**
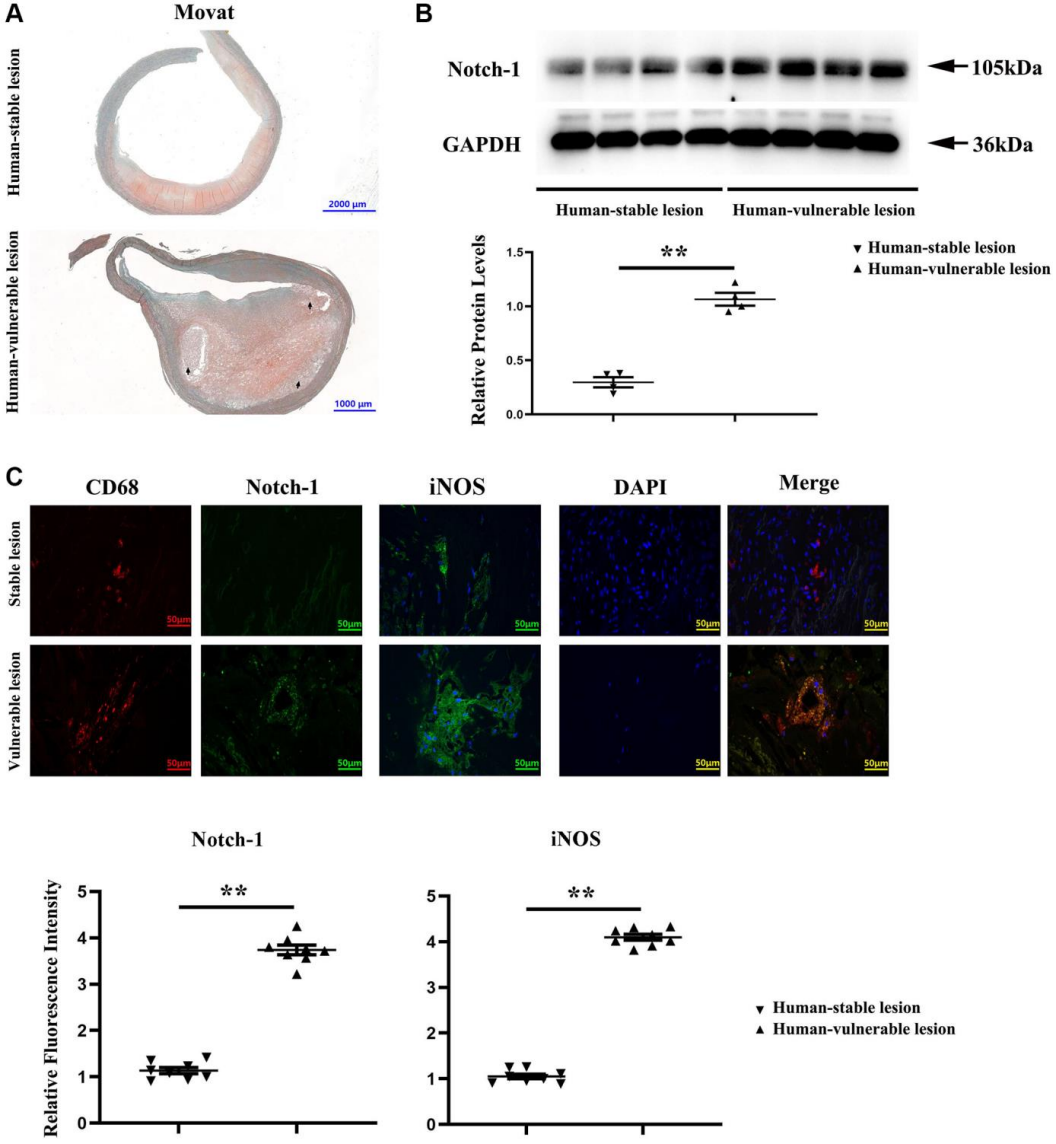
**Notch-1 was significantly increased in the human vulnerable atherosclerotic lesion.** (**A**) Representative images of the human vulnerable atherosclerotic lesion and stable lesion. In the representative images of the human vulnerable atherosclerotic lesion, thinning of fibrous caps, enlargement of lipid core and necrotic sites, and macrophage increase could be observed. (**B**) Western blotting and quantitative analysis revealed that the Notch-1 expression was significantly enhanced in the human vulnerable atherosclerotic lesion. ^**^P < 0.01: human vulnerable atherosclerotic lesion vs. stable lesion. (**C**) Immunofluorescence microscopy, and quantitative analysis revealed increased immunofluorescence of Notch-1 and iNOS in macrophages of the human vulnerable atherosclerotic lesion compared with the stable lesion. ^**^P < 0.01: human vulnerable atherosclerotic lesion vs. stable lesion.

To explore the effect of macrophage Notch-1 in human AS, the Notch-1 expression was first examined in human carotid tissues with vulnerable or stable atherosclerotic lesions. Representative Movat staining images of vulnerable atherosclerotic lesions and stable lesions in human carotid arteries are shown in Figure 1A. The intra-plaque hemorrhage, necrotic cores as well as extensive lipid deposition in the intima were found in vulnerable plaques rather than in stable plaques according to the pathological classification of previous methods [23, 24]. The results of Western blotting showed that the Notch-1 protein level was significantly increased by approximately 6–7 folds in vulnerable lesions compared with that in stable lesions (Figure 1B, *r* < 0.05). The double immunostaining results of Notch-1 and macrophages (CD68+) and iNOS (inflammatory factor) immunofluorescence monostain showed that the macrophages in vulnerable lesions expressed more abundant Notch-1 and iNOS than those in stable lesions (Figure 1C). These results showed elevated levels of Notch-1 and iNOS in macrophages of human vulnerable atherosclerotic lesion, indicating a functional role of macrophage Notch-1 in unstable AS.

### Macrophage-specific Notch-1 deletion repressed AS

**Figure 2:**
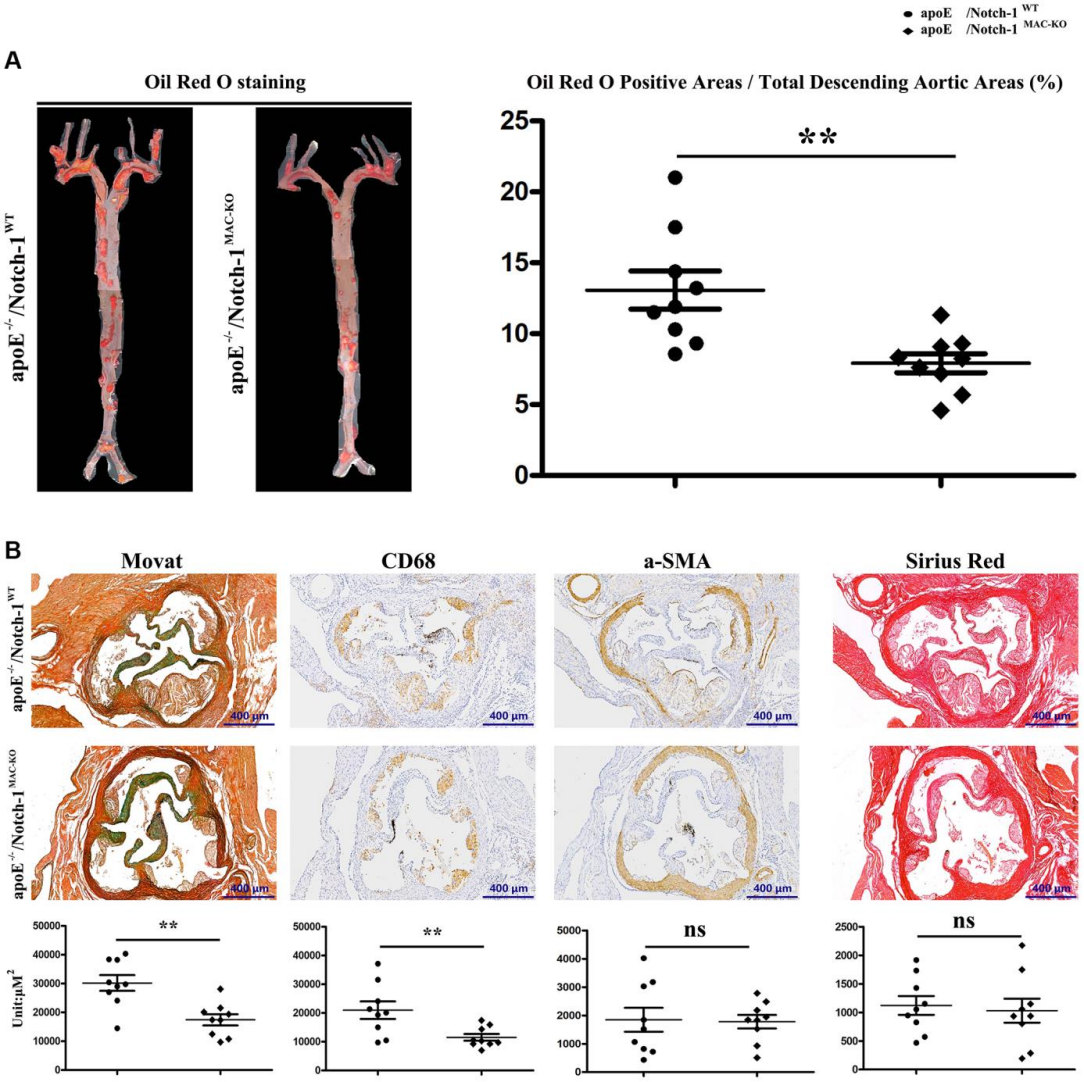
**Specific deletion of Notch-1 in macrophage repressed AS.** (**A**) Oil red O staining in thoracic aortas and quantitative analysis revealed that Notch-1^MAC-KO^ significantly decreased the atherosclerotic plaques compared with Notch-1^WT^. ^**^P < 0.01: ApoE^−/−^/Notch-1^MAC-KO^ vs. ApoE^−/−^/Notch-1^WT^. (**B**) Movat, α-SMA, CD68 and Sirius red staining and quantitative analysis revealed that Notch-1^MAC-KO^ could decrease plaque cellularity (Movat staining) and macrophage infiltration (CD 68) compared with Notch-1^WT^. However, there was no significant difference in smooth muscle cell content (α-SMA staining) and fibrotic lesions (Sirius red staining) between the two groups. ^**^P < 0.01: ApoE^−/−^/Notch-1^MAC-KO^ vs. ApoE^−/−^/Notch-1^WT^.

To examine the potential causal relationship between macrophage Notch-1 elevation and AS progression, ApoE^−/−^ mice were crossed with Notch-1^MAC-KO^ mice to generate ApoE^−/−^/Notch-1^MAC-KO^ mice for induction of AS. The littermate ApoE^−/−^/Notch-1^WT^ mice served as controls. In accordance with previous literature [25, 26], all the ApoE^−/−^ mice, regardless of the presence or absence of macrophage Notch-1 knockout, developed AS after being fed a high-fat diet. Compared with the control (W.T. group), the Notch-1^MAC-KO^ mice (K.O. group) showed a significant decrease in oil red O-positive area (Figure 2A, *r* < 0.05), indicating that AS severity is reduced by macrophage Notch-1 deletion.

The morphological features of plaques in thoracic aortas were further observed by the Movat staining, immunostaining, and Sirius red staining. As shown in Figure 2B, the plaque morphology revealed that KO exposure alleviated AS by decreasing plaque cellularity (Movat staining) and macrophage infiltration (CD68 positive) rather than the smooth muscle cell content (α-SMA positive) and fibrotic lesions (Sirius red staining). The quantitative analysis of staining results revealed that compared with the W.T. group, KO exposure significantly reduced the plaque cellularity and macrophage content (Figure 2B, *r* < 0.05). These results suggest that specific deletion of Notch-1 in macrophages attenuates AS development in high-fat diet-fed ApoE^−/−^ mice.

### Notch-1^MAC-KO^ enhanced M1-to-M2 transition and inhibited apoptosis

**Figure 3:**
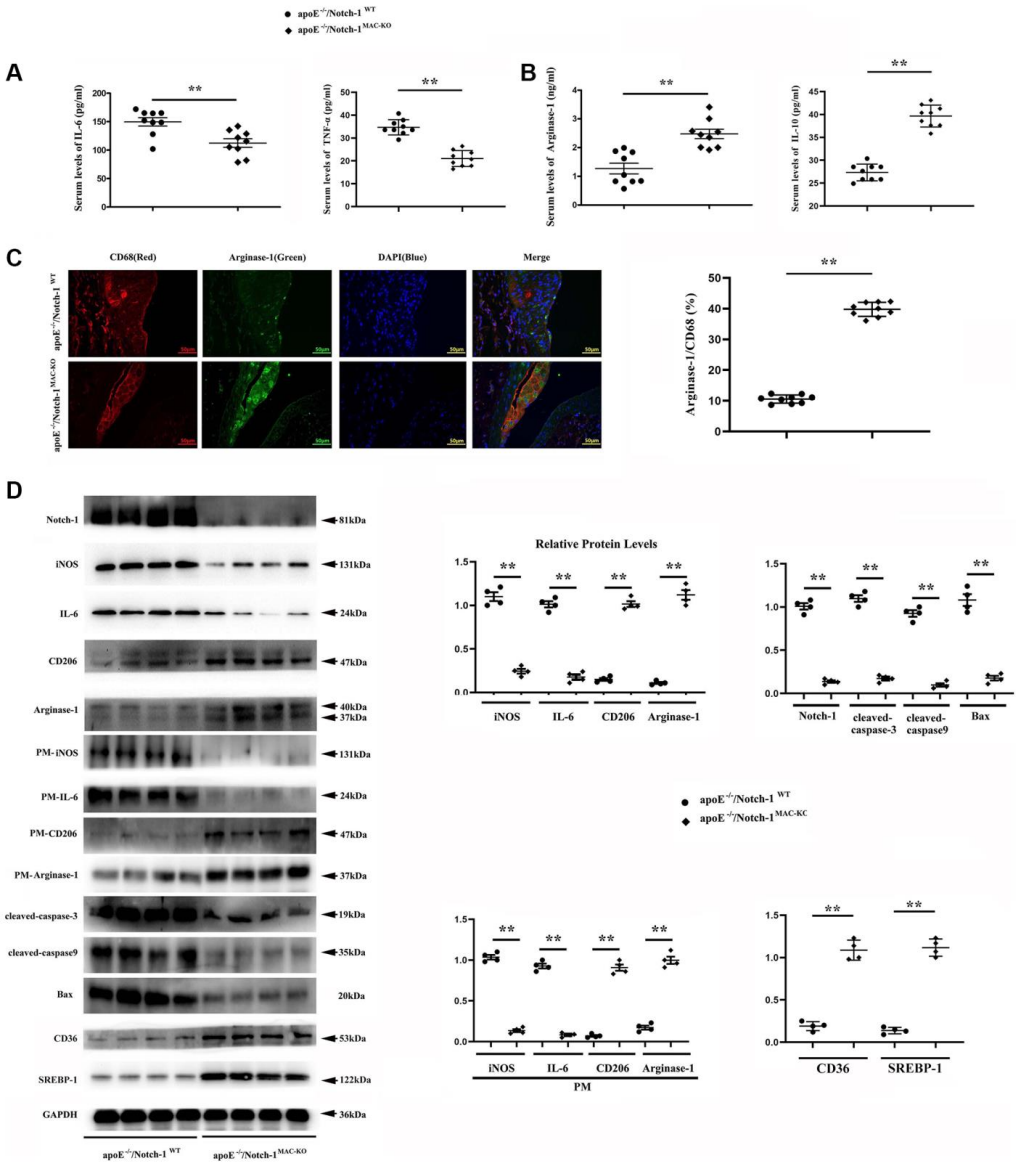
**Notch-1^MAC-KO^ repressed pro-inflammatory (M1) responses and stimulated anti-inflammatory (M2) effects in AS.** (**A**, **B**) IL-6 and TNFα (M1 markers) were decreased in K.O. mice, whereas Arg-1 and IL-10 (M2 markers) were increased in K.O. mice. (^**^P < 0.01: ApoE^−/−^/Notch-1^MAC-KO^ vs. ApoE^−/−^/Notch-1^WT^). (**C**) The aortic roots were collected, and immunofluorescent staining showed decreased staining of positive macrophages (CD68) and increased staining of Arg-1 in Notch-1^MAC-KO^ mice. (**D**) Western blotting and quantitative analysis revealed down-regulated Notch-1, IL-6 (from peritoneal macrophages), iNOS (from peritoneal macrophages), cleaved-caspase-3, caspase-9 and Bax, and up-regulated CD206, Arg-1, CD36, SREBP-1, CD206 (from peritoneal macrophages) and Arg-1 (from peritoneal macrophages) in Notch-1^MAC-KO^ mice. ^**^P < 0.01: ApoE^−/−^/Notch-1^MAC-KO^ vs. ApoE^−/−^/Notch-1^WT^.

To explore how Notch-1^MAC-KO^ affects macrophage phenotype and vascular inflammation, M1/M2 makers of macrophages, and inflammatory cytokine markers were detected. ELISA results showed that the contents of IL-6 and TNFα in the Notch-1^MAC-KO^ group were significantly reduced and the contents of IL-10 and Arg-1 were significantly increased compared to the Notch-1^WT^ group. Compared with the Notch-1^WT^ group and the Notch-1^MAC-KO^ group, the content of IL-6, TNFα, IL-10 and Arg-1 in the control group was significantly reduced (Figure 3A, 3B). The results of immunofluorescence staining showed that the CD68-positive area in atherosclerotic lesions in atherosclerotic lesions were significantly decreased in Notch-1^MAC-KO^ mice, suggesting decreased macrophage accumulation and decreased vascular inflammation. In contrast, the immunostaining for Arg-1 (an M2 marker) was increased. Furthermore, co-staining of Arg-1 and CD68 showed large amounts of Arg-1 in macrophages, which was increased by Notch-1^MAC-KO^ (Figure 3C).

Consistent with the immunostaining results, the Western blotting results showed that the protein levels of M1 markers interleukin-6 (IL-6) and iNOS were down-regulated, whereas those of M2 markers CD206, Arg-1, SREBP-1 and CD36 were up-regulated in the atherosclerotic lesions and peritoneal macrophages of Notch-1^MAC-KO^ mice. The expression of apoptosis-associated proteins cleaved-caspase-3, caspase-9 and Bax in the Notch-1^MAC-KO^ group was also significantly reduced (Figure 3D).

Taken together, these results demonstrate that Notch-1^MAC-KO^ reduces M1 and enhances M2 phenotype of macrophages, and has an anti-inflammatory effect in protecting ApoE^−/−^ mice from developing AS.

### Correlation between Notch-1 and PI3K in AS

**Figure 4:**
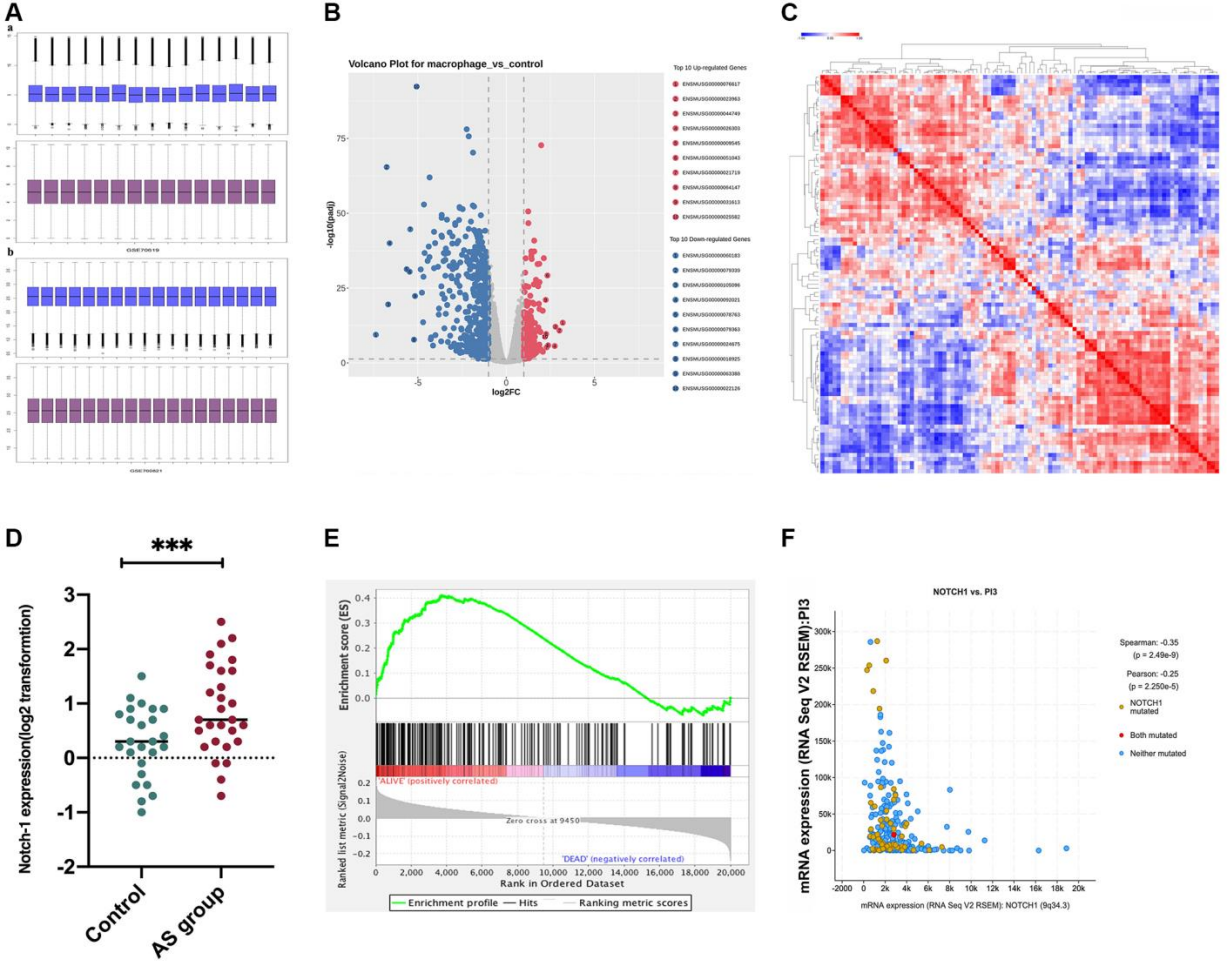
**Correlation between PI3K and Notch-1 signaling activation.** GSE155842 and GSE155745 datasets were downloaded from the GEO database, which contained AS samples. (**A**) The gene expression levels from GSE155842 were standardized using quartile division. (**B**) We identified 270 up-regulated differentially expressed genes (DEGs) and 436 down-regulated DEGs were obtained using criteria of |logFC| > 2, *P* adj < 0.05; (**C**) Based on GSE155842, the hierarchical clustering analysis exhibited a great difference in DEGs as indicated in the heat maps. (**D**) The survival analysis demonstrated that the survival time of patients with high expressions of Notch-1 was significantly shorter than those with low expressions. (**E**) The Notch-1 expression was also at a high level in the AS group. (**F**) Co-expression analysis revealed that Notch-1 was negatively associated with PI3K.

To investigate the signaling mechanism of Notch-1, the PI3K/AKT pathway was preferentially investigated, as it acts as the main pathway mediating signals from multiple receptors and plays a crucial role in macrophage polarization and AS [27]. First, bioinformatics analysis was performed to determine the association between Notch-1 and PI3K, using GSE155842 downloaded from the GEO database. GSE155842 were bone marrow-derived macrophages (BMDM) RNA-seq dataset from *Mus musculus* cell line, including stimulated macrophages AS group samples and control group samples. The gene expression levels from GSE155842 were standardized using quartile division, and the pre-standardization and post-standardization data were exhibited in Figure 4A and Supplementary Table 1. We identified 270 up-regulated differentially expressed genes (DEGs) and 436 down-regulated DEGs were obtained using criteria of |logFC| > 2, *P* adj < 0.05 (Figure 4B). Hierarchical clustering analysis exhibited an intense distinction on differentially expressed genes as indicated in the heat map (Figure 4C). Furthermore, the Notch-1 gene was expressed at a high level in the AS group (Figure 4D, *r* < 0.05). Notably, the co-expression analysis showed that the Notch-1 mRNA level showed an inverse correlation with PI3K (Figure 4F, *r* < 0.05, R < −0.3). To further assess whether Notch-1 activated genes showed statistical significance with PI3K, GSEA was carried out (Figure 4E). As shown in Figure 4F, a statistically significant association between Notch-1 activation and PI3K expression was found. In brief, these results suggest that Notch-1 gene expression is negatively correlated with PI3K in AS, indicating that Notch-1 may mediate PI3K signaling.

**Figure 5:**
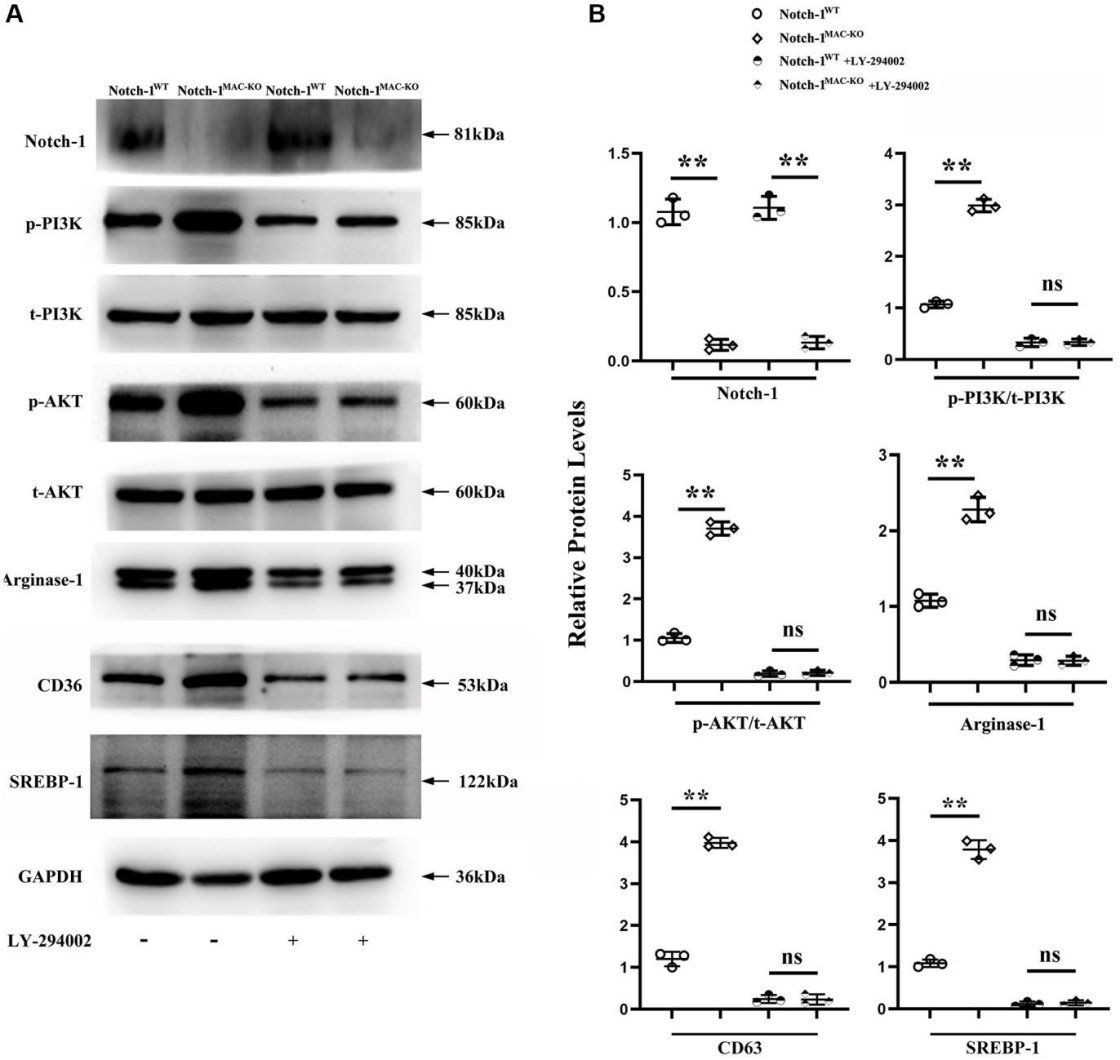
**Notch-1^MAC-KO^ reduced the secretion of PI3K and caused M2 anti-inflammatory effect.** (**A**) Western blotting revealed that the expression levels of Notch-1, p-PI3K, p-AKT, CD36, SREBP-1 and Arg-1 were markedly enhanced in the peritoneal macrophages of Notch-1^MAC-KO^ mice, which were reversed by an anti-PI3K neutralizing antibody (LY-294002). (**B**) Quantitative analysis of relative protein levels revealed that Notch-1, p-PI3K, p-AKT and Arg-1 were increased in the peritoneal macrophages of K.O. group compared with W.T. group, which were significantly reversed by LY-294002. ^**^P < 0.01, ns *P* > 0.05: peritoneal macrophages of Notch-1^MAC-KO^ mice vs. peritoneal macrophages of Notch-1^WT^ mice.

**Figure 6:**
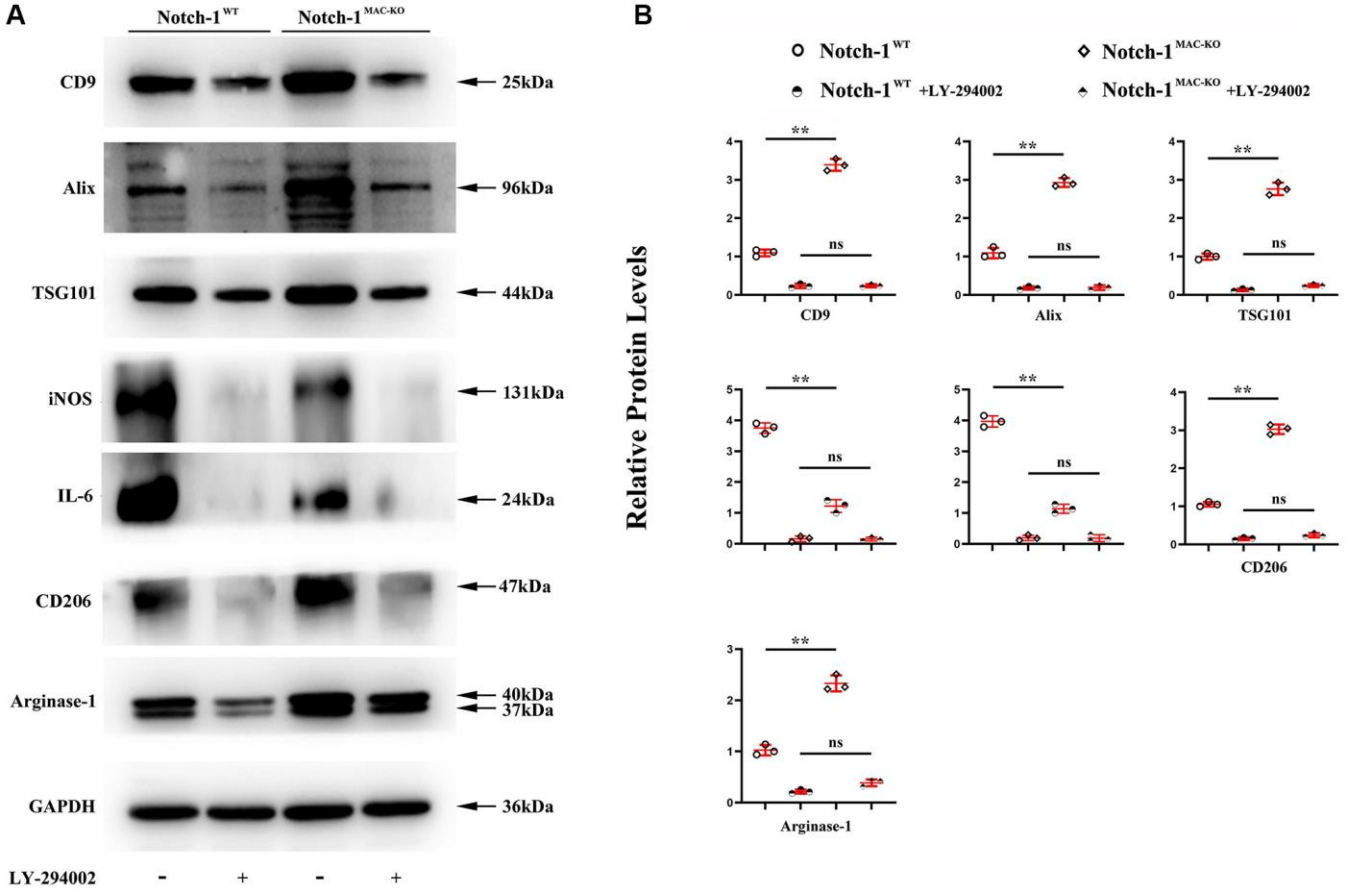
**PI3K/AKT signaling pathway was involved in the Notch-1^MAC-KO^-induced M2 polarization.** (**A**) Western blotting showed that CD9, TSG101, Alix, CD206 and Arg-1 were significantly enhanced in peritoneal macrophages of Notch-1^MAC-KO^ mice, but iNOS and IL-6 were significantly reduced, which were reversed by LY-294002. Compared with W.T. group, the expression levels of CD9, TSG101, Alix and Arg-1 were enhanced in K.O. group; (**B**) statistical data for Western blot. ^*^P < 0.05, ^**^P > 0.01: Notch-1^MAC-KO^ vs. Notch-1^WT^, Notch-1^WT^ vs. Notch-1^WT^ + LY-294002, Notch-1^MAC-KO^ vs. Notch-1^MAC-KO^ + LY-294002.

**Figure 7:**
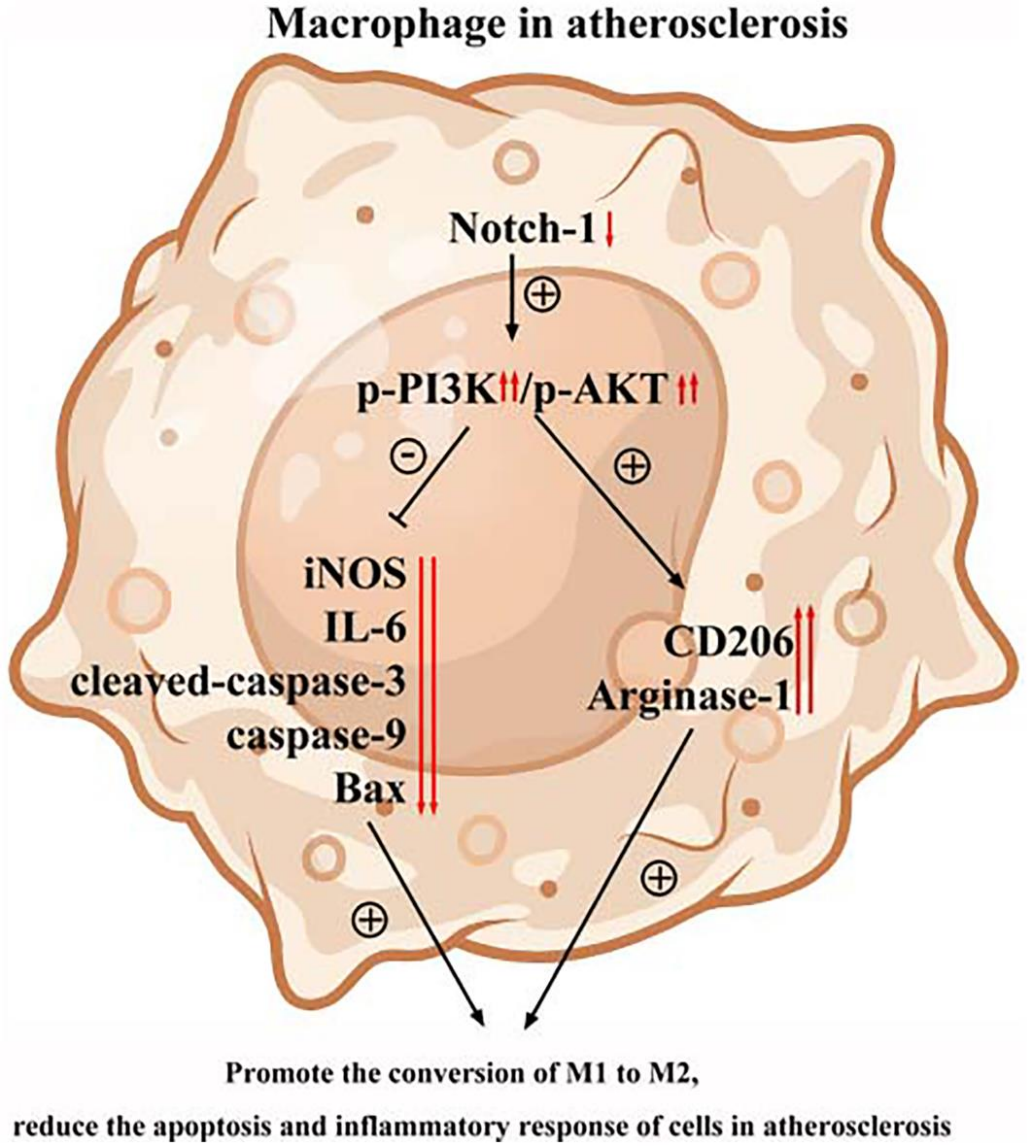
The PI3K/AKT signaling pathway was involved in the Notch-1^MAC-KO^-induced M2 polarization and further reduced apoptosis and inflammatory response in AS.

Next, an anti-PI3K neutralizing antibody LY-294002 was employed to examine the mediation role of PI3K pathway in Notch-1-derived M2 polarization. LY-294002 (10 μmol/L) was added to macrophages. As shown in Figure 5, PI3K and AKT were both inactivated in the aortic tissue of W.T. mice receiving LY-294002. Meantime, the expression of the Arg-1, an effector of PI3K/AKT activation [28], was inhibited by LY-294002. For the K.O. mice, the over-activation of PI3K and AKT was prevented, and the up-regulated expression of Arg-1, CD36, SREBP-1 reversed by LY-294002. These results suggest that Notch-1^MAC-KO^ induced M2 anti-inflammatory effects via the PI3K/AKT pathway. Considering that exosomes serve as an important means for Arg-1 delivery [29–31], the effects of Notch-1 on exosome Arg-1 secretion through the PI3K/AKT pathway were examined. Finally, whether the peritoneal macrophages of Notch-1^MAC-KO^ mice secreted more exosomes and induced M2 polarization was determined. Exosomes were isolated from the supernatant of peritoneal macrophages of Notch-1^MAC-KO^ and Notch-1^WT^ mice via ultracentrifugation. The levels of CD9, TSG101, Alix (the markers for exosomes), iNOS, IL-6, CD206, and Arg-1 were compared using Western blotting. The results showed that their expression levels were significantly enhanced in peritoneal macrophages of Notch-1^MAC-KO^ mice, which were reversed by LY-294002 (Figure 6A, 6B), suggesting that the PI3K/AKT signaling pathway is involved in and restrains M2 polarization (*r* < 0.05). Compared with the W.T. group, the expression levels of CD9, TSG101, Alix, CD206 and Arg-1 were enhanced in the K.O. group, but the expression of the M1 markers iNOS and IL-6 was reversed (Figure 6B, *r* < 0.05). To sum up, these results implied that the PI3K/AKT signaling pathway is involved in the Notch-1^MAC-KO^-induced M2 polarization and further reduces the inflammatory response in AS (Figure 7).

## DISCUSSION

In the present study, it was proved that Notch-1^MAC-KO^ successfully induced an M2 anti-inflammatory phenotype characterized by a high expression of Arg-1. Besides, Notch-1^MAC-KO^ activated the M2 anti-inflammatory effect via the PI3K signaling pathway in the repression of AS. To explain these, representative images of the human vulnerable atherosclerotic lesions and stable lesions were first presented. It was found that Notch-1 was significantly enhanced in the human vulnerable atherosclerotic lesion. The immunofluorescence microscopy revealed increased Notch-1 in the macrophages of the human vulnerable atherosclerotic lesions. To determine the causal relationship between the Notch-1 increase in macrophages and the progression of AS, the floxed Notch1 mice were crossed with mice expressing the Cre recombinase under the control of Lyz2 promoter to obtain Notch-1^MAC-KO^ mice, with their Notch-1^flox/flox^ littermates as W.T. controls. All mice fed with a high-fat diet suffered from AS. Oil red O staining was used to mark AS. By estimating the percentage of oil red O-positive areas, significant atherosclerotic plaques were found in aortas in both groups, suggesting the AS models were established successfully. Most importantly, Notch-1^MAC-KO^ significantly decreased the atherosclerotic plaques. Movat staining, α-SMA, CD68 and Sirius red staining revealed that Notch-1^MAC-KO^ could decrease plaque cellularity (Movat staining) and macrophage infiltration (CD68). However, there was no significant difference in smooth muscle cell content (α-SMA staining) and fibrotic lesions (Sirius red staining) between the two groups, suggesting that the specific deletion of Notch-1 in macrophages may inhibit the development of AS.

Given that macrophages are present in two major subsets of M1 and M2 macrophages, typically activated M1 macrophages intermediate pro-inflammatory responses, whereas alternatively activated M2 macrophages inhibit inflammatory responses [8]. In this study, it was verified that IL6 and iNOS (M1 markers) were significantly down-regulated in Notch-1^MAC-KO^ mice, whereas CD206 and Arg-1 were up-regulated in Notch-1^MAC-KO^ mice, suggesting that Notch-1^MAC-KO^ represses pro-inflammatory (M1) responses and enhances anti-inflammatory (M2) responses, and has an anti-inflammatory effect on the development of AS.

Earlier work has shown that Notch-1 knockdown increases PTEN protein levels [4], and PTEN can inhibit PI3K signaling through dephosphorylating PIP3 and producing phosphatidylinositol 4,5-bisphosphate [9]. However, the relationship between Notch-1 and PI3K signaling pathways in AS has not been investigated previously. Thus, whether there was an association between PI3K activation and Notch-1 expression in AS was detected by bioinformatics analysis. GSE155842 and GSE155745 datasets containing AS samples were downloaded from the GEO database. The gene expression levels from GSE155842 and GSE155745 were standardized using quartile division, and the pre-standardization and post-standardization data were exhibited. 670 DEGs were identified in the heat map. Furthermore, the survival analysis demonstrated that the survival time of patients with high expressions of Notch-1 was significantly shorter than those with low expressions. Notch-1 was also expressed at a high level in AS tissues (*P* < 0.05). Furthermore, it was found that Notch-1 was negatively associated with PI3K (*P* < 0.05, *R* < −0.3). GSEA revealed a statistically significant association between Notch-1 activation and PI3K expression, suggesting that Notch-1 activation is negatively associated with PI3K in AS.

*In vitro* Western blotting revealed that p-PI3K, p-AKT, and Arg-1 were markedly enhanced in peritoneal macrophages of K.O. mice, consistent with the view that the PI3K/Akt signaling pathway can stimulate activation of M2 anti-inflammatory macrophage. To further explore whether PI3K signaling was involved in the Notch-1^MAC-KO^-induced AS repression, LY-294002, an inhibitor of PI3K, was injected into the peritoneal macrophages of Notch-1^MAC-KO^ mice. It was found that the enhanced levels of p-PI3K, p-AKT, and Arg-1 were reversed by LY-294002, suggesting that PI3K is activated and associated with Notch-1^MAC-KO^-induced AS repression. The expression of apoptosis protein was detected by Western blotting, and the results showed that the expressions of cleaved-caspase-3, caspase-9 and Bax in Notch-1^MAC-KO^ mice were significantly down-regulated, indicating that knocking out Notch-1 can inhibit apoptosis in AS [32, 33]. Exosomes are endosome-derived extracellular vesicles (E.V.), transporting various substances, such as proteins, mRNA and acids to adjacent cells, which may act in a paracrine manner to make different contributions, indicating a new interaction mode between cells [10]. Arg-1, a marker for the M2 anti-inflammatory macrophages, plays a crucial role in the treatment of AS [11, 12]. Therefore, in this study, whether exosomes derived Arg-1 from macrophages could inhibit M1 polarization, promote M2 polarization, further reduce the inflammatory response and improve the regeneration of arteriosclerotic lesions was investigated. Exosomes were isolated from the supernatant of peritoneal macrophages of Notch-1^MAC-KO^ and Notch-1^WT^ mice via ultracentrifugation. The morphology of exosomes in peritoneal macrophages of AS pretreated with ox-LDL was observed by TEM. Increased oval exosome vesicles with enlarged diameter in the peritoneal macrophages of Notch-1^MAC-KO^ mice were observed compared to W.T. group. Moreover, evidence from several studies has indicated that the PI3K/AKT signaling pathway plays a role in the secretion of exosomes [34, 35]. To further explore whether Notch-1^MAC-KO^-induced increase in exosomes was achieved via the PI3K/AKT signaling pathway, LY-294002 was injected into the peritoneal macrophages of Notch-1^MAC-KO^ mice. Then it was found that such an increase was significantly reversed by LY-294002. In addition, the levels of CD9, TSG101, Alix, iNOS, IL-6, CD206, CD36, SREBP-1 and Arg-1 in exosomes were detected by Western blotting. The results showed that the expression levels of CD206 and Arg-1 in peritoneal macrophages of Notch-1^MAC-KO^ mice were significantly enhanced, while the expression levels of iNOS and IL-6 were significantly reduced, which were reversed by LY-294002, suggesting that the PI3K/AKT signaling pathway is involved in the Notch-1^MAC-KO^-induced M2 polarization.

In conclusion, this study validated the protective effect of Notch-1^MAC-KO^ against AS *in vivo* and *in vitro*. The PI3K/AKT signaling pathway is involved in the Notch-1^MAC-KO^-induced M2 polarization and further reduces apoptosis and inflammatory response in AS. The present work revealed Notch-1 as a novel therapeutic target for AS through targeting the PI3K/AKT signaling pathway. Therefore, the Notch-1/PI3K/AKT signaling pathway may serve as a novel approach for prevention and treatment of AS.

## Supplementary Materials

Supplementary Table 1

## References

[1] Baumer Y, Ng Q, Sanda GE, Dey AK, Teague HL, Sorokin AV, Dagur PK, Silverman JI, Harrington CL, Rodante JA, Rose SM, Varghese NJ, Belur AD, et al. Chronic skin inflammation accelerates macrophage cholesterol crystal formation and atherosclerosis. JCI Insight. 2018; 3:97179. 10.1172/jci.insight.97179 29321372 PMC5821196

[2] Zhao J, Xu SZ, Liu J. Fibrinopeptide A induces C-reactive protein expression through the ROS-ERK1/2/p38-NF-κB signal pathway in the human umbilical vascular endothelial cells. J Cell Physiol. 2019; 234:13481–92. 10.1002/jcp.28027 30633345 PMC13483268

[3] Ju R, Zhuang ZW, Zhang J, Lanahan AA, Kyriakides T, Sessa WC, Simons M. Angiopoietin-2 secretion by endothelial cell exosomes: regulation by the phosphatidylinositol 3-kinase (PI3K)/Akt/endothelial nitric oxide synthase (eNOS) and syndecan-4/syntenin pathways. J Biol Chem. 2014; 289:510–9. 10.1074/jbc.M113.506899 24235146 PMC3879572

[4] Rotar OP, Boyarinova MA, Moguchaia EV, Kolesova EP, Erina AM, Solntsev VN, Konradi AO, Shlyakhto EV. Subclinical target organ damage in subjects with different components of metabolic syndrome. Clin Exp Hypertens. 2018; 40:421–6. 10.1080/10641963.2017.1384488 29068233

[5] D'Onofrio N, Servillo L, Balestrieri ML. SIRT1 and SIRT6 Signaling Pathways in Cardiovascular Disease Protection. Antioxid Redox Signal. 2018; 28:711–32. 10.1089/ars.2017.7178 28661724 PMC5824538

[6] Rupprecht S, Finn S, Hoyer D, Guenther A, Witte OW, Schultze T, Schwab M. Association Between Systemic Inflammation, Carotid Arteriosclerosis, and Autonomic Dysfunction. Transl Stroke Res. 2020; 11:50–9. 10.1007/s12975-019-00706-x 31093927

[7] Jensen LD, Hot B, Ramsköld D, Germano RFV, Yokota C, Giatrellis S, Lauschke VM, Hubmacher D, Li MX, Hupe M, Arnold TD, Sandberg R, Frisén J, et al. Disruption of the Extracellular Matrix Progressively Impairs Central Nervous System Vascular Maturation Downstream of β-Catenin Signaling. Arterioscler Thromb Vasc Biol. 2019; 39:1432–47. 10.1161/ATVBAHA.119.312388 31242033 PMC6597191

[8] Angelin B, Eriksson M, Rudling M. Lipoprotein metabolism. Arterioscler Thromb Vasc Biol. 2019; 39:1699–701.31433697

[9] Soldano S, Trombetta AC, Contini P, Tomatis V, Ruaro B, Brizzolara R, Montagna P, Sulli A, Paolino S, Pizzorni C, Smith V, Cutolo M. Increase in circulating cells coexpressing M1 and M2 macrophage surface markers in patients with systemic sclerosis. Ann Rheum Dis. 2018; 77:1842–5. 10.1136/annrheumdis-2018-213648 30012643

[10] Chistiakov DA, Myasoedova VA, Revin VV, Orekhov AN, Bobryshev YV. The impact of interferon-regulatory factors to macrophage differentiation and polarization into M1 and M2. Immunobiology. 2018; 223:101–11. 10.1016/j.imbio.2017.10.005 29032836

[11] Momtazi-Borojeni AA, Abdollahi E, Nikfar B, Chaichian S, Ekhlasi-Hundrieser M. Curcumin as a potential modulator of M1 and M2 macrophages: new insights in atherosclerosis therapy. Heart Fail Rev. 2019; 24:399–409. 10.1007/s10741-018-09764-z 30673930

[12] Siebel C, Lendahl U. Notch Signaling in Development, Tissue Homeostasis, and Disease. Physiol Rev. 2017; 97:1235–94. 10.1152/physrev.00005.2017 28794168

[13] Liu ZJ, Li Y, Zhang L, Regueiro MM, Wei Y, Velazquez OC. Abstract 224: Notch1 Activation in Endothelial Cells Promotes Progression of Atherosclerosis. Arterioscler Thromb Vasc Biol. 2018; 38:A224. 10.1161/atvb.38.suppl_1.224

[14] Singla DK, Wang J, Singla R. Primary human monocytes differentiate into M2 macrophages and involve Notch-1 pathway. Can J Physiol Pharmacol. 2017; 95:288–94. 10.1139/cjpp-2016-0319 28238274

[15] Vergadi E, Ieronymaki E, Lyroni K, Vaporidi K, Tsatsanis C. Akt Signaling Pathway in Macrophage Activation and M1/M2 Polarization. J Immunol. 2017; 198:1006–14. 10.4049/jimmunol.1601515 28115590

[16] Calzavara E, Chiaramonte R, Cesana D, Basile A, Sherbet GV, Comi P. Reciprocal regulation of Notch and PI3K/Akt signalling in T-ALL cells in vitro. J Cell Biochem. 2008; 103:1405–12. 10.1002/jcb.21527 17849443

[17] Hales EC, Taub JW, Matherly LH. New insights into Notch1 regulation of the PI3K-AKT-mTOR1 signaling axis: targeted therapy of γ-secretase inhibitor resistant T-cell acute lymphoblastic leukemia. Cell Signal. 2014; 26:149–61. 10.1016/j.cellsig.2013.09.021 24140475

[18] Jin Y, Li C, Xu D, Zhu J, Wei S, Zhong A, Sheng M, Duarte S, Coito AJ, Busuttil RW, Xia Q, Kupiec-Weglinski JW, Ke B. Jagged1-mediated myeloid Notch1 signaling activates HSF1/Snail and controls NLRP3 inflammasome activation in liver inflammatory injury. Cell Mol Immunol. 2020; 17:1245–56. 10.1038/s41423-019-0318-x 31673056 PMC7784844

[19] Movat HZ. Demonstration of all connective tissue elements in a single section; pentachrome stains. AMA Arch Pathol. 1955; 60:289–95. 13248341

[20] Moss ME, DuPont JJ, Iyer SL, McGraw AP, Jaffe IZ. No Significant Role for Smooth Muscle Cell Mineralocorticoid Receptors in Atherosclerosis in the Apolipoprotein-E Knockout Mouse Model. Front Cardiovasc Med. 2018; 5:81. 10.3389/fcvm.2018.00081 30038907 PMC6046374

[21] Hayes EM, Tsaousi A, Di Gregoli K, Jenkinson SR, Bond AR, Johnson JL, Bevan L, Thomas AC, Newby AC. Classical and Alternative Activation and Metalloproteinase Expression Occurs in Foam Cell Macrophages in Male and Female ApoE Null Mice in the Absence of T and B Lymphocytes. Front Immunol. 2014; 5:537. 10.3389/fimmu.2014.00537 25389425 PMC4211548

[22] Russell HK Jr. A modification of Movat's pentachrome stain. Arch Pathol. 1972; 94:187–91. 4114784

[23] Li T, Li X, Zhao X, Zhou W, Cai Z, Yang L, Guo A, Zhao S. Classification of human coronary atherosclerotic plaques using ex vivo high-resolution multicontrast-weighted MRI compared with histopathology. AJR Am J Roentgenol. 2012; 198:1069–75. 10.2214/AJR.11.6496 22528895

[24] Badimon L, Vilahur G. Thrombosis formation on atherosclerotic lesions and plaque rupture. J Intern Med. 2014; 276:618–32. 10.1111/joim.12296 25156650

[25] Emini Veseli B, Perrotta P, De Meyer GRA, Roth L, Van der Donckt C, Martinet W, De Meyer GRY. Animal models of atherosclerosis. Eur J Pharmacol. 2017; 816:3–13. 10.1016/j.ejphar.2017.05.010 28483459

[26] Rotllan N, Price N, Pati P, Goedeke L, Fernández-Hernando C. microRNAs in lipoprotein metabolism and cardiometabolic disorders. Atherosclerosis. 2016; 246:352–60. 10.1016/j.atherosclerosis.2016.01.025 26828754 PMC5357236

[27] Linton MF, Moslehi JJ, Babaev VR. Akt Signaling in Macrophage Polarization, Survival, and Atherosclerosis. Int J Mol Sci. 2019; 20:2703. 10.3390/ijms20112703 31159424 PMC6600269

[28] Rocher C, Singla DK. SMAD-PI3K-Akt-mTOR pathway mediates BMP-7 polarization of monocytes into M2 macrophages. PLoS One. 2013; 8:e84009. 10.1371/journal.pone.0084009 24376781 PMC3869858

[29] Zhang H, Liu J, Qu D, Wang L, Wong CM, Lau CW, Huang Y, Wang YF, Huang H, Xia Y, Xiang L, Cai Z, Liu P, et al. Serum exosomes mediate delivery of arginase 1 as a novel mechanism for endothelial dysfunction in diabetes. Proc Natl Acad Sci U S A. 2018; 115:E6927–36. 10.1073/pnas.1721521115 29967177 PMC6055191

[30] Zwager MC, Bense R, Waaijer S, Qiu SQ, Timmer-Bosscha H, de Vries EGE, Schröder CP, van der Vegt B. Assessing the role of tumour-associated macrophage subsets in breast cancer subtypes using digital image analysis. Breast Cancer Res Treat. 2023; 198:11–22. 10.1007/s10549-022-06859-y 36622544 PMC9883348

[31] Qin Y, Huo Z, Song X, Chen X, Tian X, Wang X. mir-106a regulates cell proliferation and apoptosis of colon cancer cells through targeting the PTEN/PI3K/AKT signaling pathway. Oncol Lett. 2018; 15:3197–201. 10.3892/ol.2017.7715 29435057 PMC5778839

[32] Chen WN, Guo SN, Wang JY, Jia LQ, Li DY, Tian Y. [Correlation between autophagy and polarization of macrophages in atherosclerosis plaque in arteriosclerosis obliterans amputees]. Yao Xue Xue Bao. 2016; 51:68–74. 27405164

[33] Kambara K, Ohashi W, Tomita K, Takashina M, Fujisaka S, Hayashi R, Mori H, Tobe K, Hattori Y. In vivo depletion of CD206+ M2 macrophages exaggerates lung injury in endotoxemic mice. Am J Pathol. 2015; 185:162–71. 10.1016/j.ajpath.2014.09.005 25447055

[34] Trombetta AC, Soldano S, Contini P, Tomatis V, Ruaro B, Paolino S, Brizzolara R, Montagna P, Sulli A, Pizzorni C, Smith V, Cutolo M. A circulating cell population showing both M1 and M2 monocyte/macrophage surface markers characterizes systemic sclerosis patients with lung involvement. Respir Res. 2018; 19:186. 10.1186/s12931-018-0891-z 30249259 PMC6154930

[35] Zhou K, Zhang T, Fan Y, Serick, Du G, Wu P, Geng D. MicroRNA-106b promotes pituitary tumor cell proliferation and invasion through PI3K/AKT signaling pathway by targeting PTEN. Tumour Biol. 2016; 37:13469–77. 10.1007/s13277-016-5155-2 27465551

